# Finite element analysis and *in vitro* tests on endurance life and durability of composite bone substitutes

**DOI:** 10.3389/fbioe.2024.1417440

**Published:** 2024-09-05

**Authors:** Amir Abbas Seraji, Reza Nahavandi, Amir Kia, Ahad Rabbani Doost, Vahid Keshavarz, Fariborz Sharifianjazi, Ketevan Tavamaishvili, Dorna Makarem

**Affiliations:** ^1^ Department of Mechanical and Industrial Engineering, University of Toronto, Toronto, ON, Canada; ^2^ Department of Polymer Engineering and Color Technology, Amirkabir University of Technology, Tehran, Iran; ^3^ Department of Biochemical and Pharmaceutical Engineering, School of Chemical Engineering, College of Engineering, University of Tehran, Tehran, Iran; ^4^ Department of Mechanical Engineering, University of Guilan, Rasht, Iran; ^5^ Department of Biomaterials, Iran Polymer and Petrochemical Institute, Tehran, Iran; ^6^ Department of Materials Engineering, Faculty of Engineering and Technology, Imam Khomeini International University, Qazvin, Iran; ^7^ Center for Advanced Materials and Structures, School of Science and Technology, The University of Georgia, Tbilisi, Georgia; ^8^ School of Medicine, Georgian American University, Tbilisi, Georgia; ^9^ Escuela Tecnica Superior de Ingenieros de Telecomunicacion Politecnica de Madrid, Madrid, Spain

**Keywords:** endurance life, durability, cyclic loading, bone constructs, fatigue strength, bone tissue engineering

## Abstract

Bone structures facilitate the regeneration and repair of bone tissue in regions where it has been damaged or destroyed, either temporarily or permanently. Therefore, the bone’s fatigue strength and durability are crucial to its efficacy and longevity. Several variables, such as the construct’s material qualities, design, and production procedure, loading and unloading cycles, and physiological conditions influence the endurance life of bone constructs. Metals, ceramics, and polymers are all routinely utilized to create bone substitutes, and each of these materials has unique features that might affect the fatigue strength and endurance life of the final product. The mechanical performance and capacity to promote bone tissue regeneration may be affected by the scaffold’s design, porosity, and pore size. Researchers employ mechanical testing under cyclic loading circumstances as one example of an experimental approach used to assess bone construction endurance. These analyses can give us important information about the stress-strain behavior, resistance to multiple loading cycles, and fatigue strength of the new structure. Predicting the endurance life of the developed construct may also be possible with the use of simulations and numerical analyses. Hence, in order to create reliable and efficient constructs for bone tissue engineering, it is crucial to understand their fatigue strength and durability. The purpose of this study is to analyze the effective parameters for fatigue strength of bone structures and to gather the models and evaluations utilized in endurance life assessments.

## 1 Introduction

In tissue engineering (TE), scaffold materials or biocompatible constructions serve as a foundation for the development of new tissue (Dorozhkin, 2015). New tissue is grown in place of injured or missing tissue by cultivating cells on these constructs and feeding them nutrition and growth factors (Esmaeili et al., 2023). Organ failure, musculoskeletal diseases, and trauma injuries are just some of the multiple ailments that might benefit from the adoption of functional tissue replacements, which is why TE was developed (Li et al., 2021; Ameri et al., 2023). Scaffolds for fresh bone development are 3D structures, and metals, ceramics, polymers, and composites, along with natural materials, are common examples of biocompatible and bone-regenerating materials used (Seraji and Bajgholi, 2022; Chen and Li, 2022). Medical techniques like bone grafting make use of bone scaffolds to aid in the healing and restoration of injured bones (Niu et al., 2023). Scaffolds may support cells structurally and let in the fluids and nutrients that are critical for cell development and tissue regeneration (Malekshahi et al., 2018; Dixon and Gomillion, 2022).

Multiple crucial factors influence bone scaffolding performance, including but not limited to structural and interior design, mechanical properties, material biocompatibility and non-toxicity, osteoinduction, and osteoconduction (Wu et al., 2022). Key mechanical performance aspects in constructing bone structures for TE applications include total strength under compression, modulus of elasticity, fatigue performance, creep behavior, and crack formation (Mirzaali et al., 2022).

Fatigue behavior occurs when a material is subjected to repeated stress or cyclic loading over an extended length of time (Putra et al., 2023). In engineered materials, including metals, polymers, ceramics, and composites, this is a typical occurrence that may lead to the initiation of cracks, the propagation of fractures, and eventually the collapse of the component (Kedir and Lemu, 2023; Mousavinasab et al., 2016; Zhang et al., 2023). Repeated application of cyclic loads or stress levels leads to plastic deformation and the development and propagation of tiny fractures in a component or structure (Wang et al., 2023). A steady spread of these fissures may cause extensive damage and the ultimate collapse of the structure.

The fatigue performance and durability of bone substitutes made by various manufacturing technologies have been examined through multiple studies in recent years, while their summarized results suggest that a rough surface is the principal cause of fracture initiation and, accordingly, lowering the endurance limit (Fang et al., 2022; Bakhtiari et al., 2023). Determining the connected pore network for promoting vascularization and the stable mechanical properties of an implanted bone construct lets you guess how long the structure will last (Li et al., 2016; Adekoya et al., 2023). It is also essential to investigate the mechanical response and fatigue performance of the porous scaffold (Tavakolinejad et al., 2023).

Researchers are trying to investigate a material’s fatigue behavior and endurance life by putting it through a series of fatigue tests to see how many cycles it can tolerate before breaking (Jasemi et al., 2022). Recent studies suggest that developing bone constructs from robust and long-lasting materials utilizing efficient manufacturing strategies will minimize fatigue failure in crucial structures, considering the fatigue behavior of materials (Elahinia et al., 2016; Song et al., 2023; Behseresht and Park, 2024).

This review discusses several aspects of the fatigue behavior and durability of bone substitutes, considering both theoretical and experimental methods. The role of natural and synthetic polymers in bone tissue engineering, and their various strengthen strategies have been reviewed. In addition, effective criteria for extending the endurance life of bone constructs are studied.

## 2 Prolonging endurance life and durability

Bone substitutes are often utilized to assist and direct the formation of new bone tissue in TE (Christy et al., 2020). Since the constructs must be robust enough to endure the mechanical stresses and strains placed on them throughout the healing process, their mechanical properties are critical for efficient bone regeneration (Beheshtizadeh et al., 2022). A bone scaffold’s mechanical endurance limit is critical since it is the maximum stress that will not cause the scaffold to break after a specified number of loading cycles (Zhao et al., 2023). In a fatigue test, for example, the endurance limit is determined by subjecting the scaffold to a series of loading and unloading cycles until it fails (Bakhtiari et al., 2023). The fatigue limit of a bone construct may be calculated by determining the minimum number of loading cycles at which failure occurs.

As a major goal of tissue engineering (TE), developing suitable substitutes to accelerate the bone regeneration process could be further enhanced if the design and manufacturing of the scaffold are based on the patient’s specific conditions (Ghanad et al., 2023; Soleymani et al., 2023). Considering that the mechanical properties of bones vary under different physiological conditions, it is essential to choose the optimal scaffold parameters, including material, manufacturing technology, interior and exterior architecture, physical and biological performance, and osteogenic potential (Yu et al., 2022).

When designing and selecting suitable scaffolds for bone regeneration, understanding the durability limit of a bone scaffold is essential. Structures having a higher endurance limit are more suited to load-bearing applications, such as bone regeneration in weight-bearing bones, since they can resist greater mechanical loads and strains (Entezari et al., 2020). Scaffolds having lower endurance limits, on the other hand, may be more suited to non-load-bearing applications, such as minor bone deformity correction. Bone scaffolds, irrespective of the material, may be changed and their endurance limit enhanced by including the properties described in Table 1.

**TABLE 1:** Items affecting the endurance life of bone constructs.

No.	Item	Explanation	Reference
1	Materials used in fabricating bone constructs	Metals, ceramics, polymers, hydrogels, and composites are used for scaffolding, while each of them varies in properties and features	Putra et al. (2023)
2	Scaffold porosity	The pores’ amount and size, along with their size distribution, could be various, depending on the materials and manufacturing process. Generally, higher porosity diminishes endurance	Baptista and Guedes (2021)
3	Interior structure and Interconnectivity	Interconnectivity is defined as the degree of connection among pores in the developed bone substitutes. Generally, higher interconnectivity could enhance the endurance life and the construct’s capability to resist fatigue	Hou et al. (2022)
4	Exterior structure and geometries	The shape, geometry, and dimensions of the developed bone substitutes could affect the load distribution on the structure and control the stress concentration	Yu et al. (2020)
5	Surface-depended performance	The surface of the developed bone constructs usually affects the implant’s success. Parameters like chemical composition and surface roughness are affecting cell attachment and the interaction with surrounding tissues	Dave and Gomes (2019)
6	The amplitude and conditions of load	Type, amplitude, frequency, and conditions of mechanical loading on the developed bone constructs could affect the endurance life of the bone constructs	Ladner et al. (2022)
7	Synthesizing and manufacturing techniques	The techniques that are used for synthesizing the powders, hydrogels, polymers, etc. Are as essential as the techniques used for manufacturing the scaffolds	Laird et al. (2021)

### 2.1 Synthesis, manufacturing, and treatment techniques

The fatigue performance and endurance limit of bone substitutes depend on the synthesis protocols of the raw materials utilized, as well as the manufacturing and treatment processes used (Ahmadi et al., 2022). Widely used primary substances in developing bone scaffolds include ceramics, polymers, metals, and composites. Moreover, the synthesis procedures include a range of techniques, including sol-gel, precipitation, sintering, electrospinning, and 3D printing (Harish et al., 2023; Ghanad et al., 2023).

In ceramics, the synthesis process involves the combination of powders, shaping the material, and then exposing it to high temperatures in order to produce densification (Dadkhah et al., 2023; Akrami et al., 2023). The selection of synthesis parameters, including temperature, duration, and environment, has a vital impact on the microstructure and mechanical characteristics of the ceramic bone replacement (Asar et al., 2020).

Polymers are synthesized using procedures such as polymerization, cross-linking, and mixing. The selection of monomers, initiators, and reaction conditions significantly impacts the mechanical strength, flexibility, and breakdown rate of the polymer (Balla et al., 2021). Natural polymers such as chitin, chitosan, collagen, alginate, hyaluronic acid, gelatin, and etc. (Guo et al., 2021) are used along with synthetic polymers, such as polyvinyl alcohol (PVA), Polylactic acid (PLA), poly glycolic acid (PGA), poly (lactic-co-glycolic) acid (PLGA), Polyethylene glycol (PEG), and etc. in bone TE applications (Javid-Naderi et al., 2023). Although natural and synthetic polymers are recognized as most usage materials in bone scaffolding, it is notable that they should strengthen before final usage.

Recalling that natural polymers are less resistant in mechanical loading and stress compared to the synthetic polymers, it is essential to enhance its performance. Previous studies showed that blending the natural and synthetic polymers is an available option for enhancing mechanical performance of structures, designed and manufactured for bone scaffolding (Zou et al., 2019; Sionkowska, 2011). While this technique could be effective in improving the physical and mechanical properties of natural polymers, but mineralization could not be occurred. Generally, to enhance the mechanical performance of natural polymers, especially for applications like bone scaffolding, several strategies can be employed.

Combining natural polymers with synthetic polymers or other reinforcing materials can significantly improve mechanical properties. For instance, hybrid natural fiber-reinforced polymer composites have shown enhanced strength and durability (Nurazzi et al., 2021). Also, treating natural fibers with chemicals can improve their adhesion to the polymer matrix, increasing strength and reducing water absorption. This can lead to better mechanical performance and durability.

Incorporating nanomaterials such as carbon nanotubes or graphene can enhance the mechanical properties of natural polymers. These nanomaterials can improve tensile strength, thermal stability, and overall durability (Wong et al., 2021). Incorporating nanomaterials into natural polymers can significantly enhance their mechanical properties, making them more suitable for demanding applications. Carbon Nanotubes (CNTs), Graphene, nano clays-which are layered mineral silicates that can improve the barrier properties, thermal stability, and mechanical strength of polymers-and metal oxide nanoparticles, including titanium dioxide and zinc oxide, which can enhance UV resistance, mechanical strength, and thermal stability (Baig et al., 2021).

Regarding the mechanisms of enhancement, it could be claimed that three primary mechanisms are involved as reinforcement, interfacial interaction, and thermal stability. Nanomaterials act as reinforcing agents, distributing stress more evenly throughout the polymer matrix. This leads to improved tensile strength and durability. Also, the large surface area of nanomaterials allows for better interaction with the polymer matrix, improving adhesion and mechanical properties. Nanomaterials can enhance the thermal stability of polymers by acting as heat sinks, thereby improving the material’s resistance to thermal degradation.

On the other hand, using bioinspired polymers, such as those mimicking the structure of natural materials like mussel-inspired catechol chemistry, can improve mechanical properties and durability (Omidian et al., 2023). Also, functionalizing natural polymers with specific chemical groups can enhance their mechanical properties and compatibility with synthetic polymers. This can lead to better performance in composite materials (Nurazzi et al., 2021). These solutions can help overcome the limitations of natural polymers, making them more suitable for demanding applications like bone scaffolding.

In addition to the polymers, metals used in bone replacements are often produced using casting, powder metallurgy, or additive manufacturing methods (Yamanoglu et al., 2021). The mechanical characteristics and biocompatibility of the metal implant are influenced by multiple synthesis factors, including alloy composition, heat treatment, and post-processing (Dinita et al., 2023). Manufacturing bone structures from composite materials is applicable, employing various techniques depending on the material composition.

Manufacturing processes dictate the overall composition and design of bone replacements. Typical manufacturing techniques are molding, 3D printing, and electrospinning. Molding procedures include the process of transforming the raw materials into the intended shape by using molds (Altan et al., 2001). This procedure is applicable to ceramics, polymers, and composites. The molding parameters, including pressure, temperature, and cooling rate, have an essential influence on the density, porosity, and mechanical characteristics of the bone replacement (Rodriguez-Contreras et al., 2021).

The use of 3D printing in the production of biomedical facilities, including biosensors and medical equipment, has attracted considerable interest, while its usage in developing tissue scaffolds and specifically bone substitutes is our focus in this content. It provides the ability to exert meticulous control over the internal composition and permeability, thereby facilitating the production of implants tailored to individual patients (Wang et al., 2021). The mechanical characteristics and pore architecture of the bone replacement are influenced by the printing parameters, including layer thickness, printing speed, and material composition (Zhang et al., 2018). 3D printing methods via their various forms, including but not limited to extrusion-based 3D printing (robocasting), selective laser sintering (SLS), and fused deposition modeling (FDM), are applicable to develop hydrogel-, metal-, and polymer-based scaffolds and their composites with ceramic materials (Jurczak and Lach, 2023), as they are applicable in other various fields.

Also, electrospinning is a method used to produce fibrous scaffolds; the process involves the placement of polymer fibers onto a collector (Brown et al., 2016). The electrospinning parameters, including polymer concentration, voltage, and distance between the collector and the spinneret, have an impact on the diameter, alignment, and mechanical characteristics of the scaffold fibers.

However, treatment procedures are also used to improve the mechanical characteristics, biocompatibility, and bioactivity of bone substitutes (Kumar et al., 2020). Conventional treatment methods include heat treatment, surface modification, and coating deposition. Heat treatment involves exposing the bone substitutes to carefully regulated cycles of heating and cooling (Unune et al., 2022). This procedure has the potential to enhance the crystallinity, phase composition, and mechanical strength of the material (Li et al., 2024).

Surface modification methods, such as plasma treatment, chemical etching, and hydrothermal treatment, modify the surface characteristics of the bone replacement (Nobles et al., 2021). These alterations may augment cell adhesion, facilitate bone ingrowth, and increase the overall biocompatibility of the implant (Foroushani et al., 2022). Some coating deposition methods, such as physical vapor deposition (PVD) and plasma spraying, are used to provide bioactive coatings onto the surface of bone substitutes (Kravanja and Finšgar, 2022; Huang et al., 2022). These coatings have the ability to facilitate the integration of bone with the implant, release chemicals that promote biological activity, and enhance the overall long-term functionality of the implant.

The mechanical performance of bone scaffolds may be significantly influenced by surface features such as roughness and surface chemistry (Dave and Gomes, 2019). An increase in surface roughness may enhance the coefficient of friction, resulting in enhanced mechanical stability (Zhou et al., 2023). Surface treatments, such as coatings or modifications to the surface’s texture, might change the way the scaffold interacts with other materials, thereby improving its resistance to fatigue and increasing its endurance limit (Pesode and Barve, 2023). Enhancing the roughness of the scaffold’s surface may facilitate the development of bone into it, thereby enhancing stability and lowering the likelihood of fatigue-induced failure.

### 2.2 Design and geometry of bone scaffold

The structural characteristics of a bone scaffold, including the dimensions and configuration of its pores, may greatly influence the way stresses are distributed inside the scaffold (Ali et al., 2020). Improving the design of the scaffold may result in a more effective distribution of material, less concentration of stress, and improved resistance to fatigue (Alaneme et al., 2022; Seraji et al., 2022). The scaffold’s pore size plays a critical role in facilitating the infiltration of cells and extracellular matrix, as well as the exchange of nutrients and waste (Wang et al., 2023). Moreover, the morphology of the pores may influence both the structural integrity of the scaffold and the arrangement of cells and matrix materials inside it. By optimizing the scaffold’s structure, its mechanical stability may be enhanced, stress concentrations can be avoided, and the likelihood of fractures and collapses can be decreased (Xue et al., 2023). An appropriately engineered scaffold may endure several loading and unloading cycles without experiencing any damage.

**FIGURE 1:**
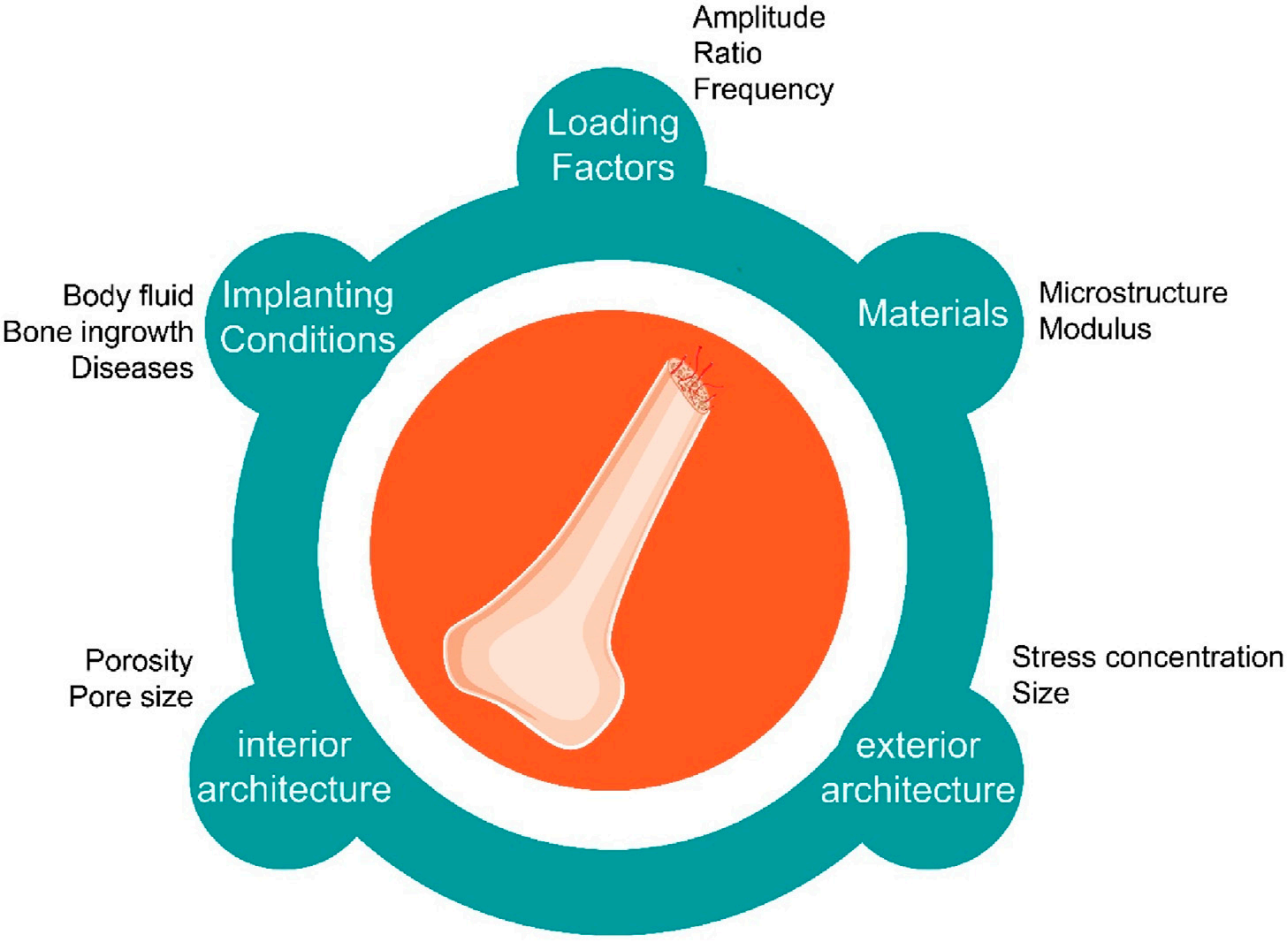
The most crucial factors in fatigue strength of bone scaffolds, including but not limited to loading factors, materials, implanting conditions, interior and exterior architecture.

The optimal geometric characteristics of bone scaffolds, such as pore size and shape, are contingent upon the particular application and materials used (Kennedy et al., 2023). Nevertheless, there exist overarching principles that may augment the endurance threshold and fatigue resistance of bone scaffolds. It is crucial to optimize the scaffold’s porosity, which represents the proportion of air space inside its walls (Sousa et al., 2023). Increased porosity promotes cell infiltration and nutrition transport, while excessive porosity may impair mechanical integrity. Hence, it is essential to achieve a harmonious equilibrium between permeability and durability. Figure 1 shows the most crucial factors in bone scaffolding fatigue strength.

The mechanical performance of the scaffold is significantly affected by the size and shape of its pores (Zheng et al., 2021). The size of the pores impacts the distribution of stress, the absorption of nutrients, and the infiltration of cells. Ideally, bone scaffolds should have a pore size ranging from 100 to 500 µm to maximize nutrition and cell transport (Jodati et al., 2020). Pores with a spherical or cylindrical shape that are interconnected with other pores promote better cell adhesion, proliferation, and even pressure distribution (Xu et al., 2021). Furthermore, the interconnected pore network is crucial for effective stress dispersion and the diffusion of nutrients and waste products throughout the scaffold (Benam et al., 2024). Maximizing pore interconnectivity ensures uniform stress distribution within the scaffold.

Designing bone scaffolds to maximize endurance limit and fatigue strength involves considering various criteria, such as intended use, biological compatibility, and mechanical demands (Bakhtiari et al., 2023). The effective Young’s modulus model is a commonly used equation that takes into account how pore shape and size influence the mechanical properties of the scaffold (Gryko et al., 2022). Equation 1 provides the formula for this calculation.

$$E_{eff}=f\times E_{matrix}\times \frac{Pore}{Pore+matrix}$$ (1)

The effective modulus of a bone scaffold, denoted as $E_{eff}$ , can be calculated using Equation 4. This equation takes into account the shape factor (f), the Young’s modulus of the matrix ( $E_{matrix}$ ), and the volume fractions of pores and matrix (pore and matrix, respectively) (Foroughi and Razavi, 2022). Determining the porosity of a bone scaffold is an important aspect of its design, and it can be calculated using Equation 2.

$$Porosity=\left[1-\frac{\text{Matrix}\;\text{Volume}}{\text{Total}\;\text{Scaffold}\;\text{Volume}}\right]\times 100$$ (2)

## 3 Finite element analysis of bone substitutes

Material qualities, design, and manufacturing procedures all influence the fatigue behaviors of materials (Zadpoor, 2019). The finite element analysis (FEA) analysis of bone constitutes is a computer-based modeling approach used to assess their structural behavior and endurance life (Abdullah, 2020). Finite elements are used to break down the scaffold into more manageable chunks. To forecast the fatigue performance and endurance life of the bone substitutes under varying loading circumstances, researchers study each component separately (Zhao et al., 2021). Computer models of the scaffolding can be used to guess its mechanical properties, how damage will be caused, and when it will finally break from overuse (Hedayati et al., 2016; Gheibi et al., 2017).

Bone substitute models are reconstructed using 3D images of their internal porosity and microarchitecture to characterize their shape and material qualities (Melancon et al., 2017). Elastic modulus, yield strength, and fatigue limit are only a few of the material parameters specified by reference to experimental data (Pang et al., 2014). The model is then made to simulate the actual physiological loading environment by defining the type, magnitude, and frequency of the loading circumstances, usually by FEA.

Several damage models may mimic the fatigue that builds up in the construction material after repeated loading and unloading (Jasemi et al., 2022). Fatigue crack propagation and eventual failure may be predicted with the use of damage mechanics models like the Paris Law (Zhou et al., 2023). Charts and figures show the mechanical reaction and internal damage to the structure. Researchers use the results to better understand how fatigue damage builds up inside the scaffold material and how to develop a scaffold that is resistant to fatigue failure (Xiong et al., 2020).

A bone substitute’s expected behavior may be better predicted by modeling fatigue behavior before actual testing (Hedayati et al., 2016). It is a cheap way to figure out whether a bone substitute can take the weight you want it to for the long haul. The results of the simulation show how small changes in design or different choices of materials can affect how well a structure holds up over time. This information can be used to promote the best-engineered solutions that have the highest fatigue resistance and overall structure effectiveness (Ryan et al., 2009). Insights on how to improve the performance and durability of scaffolds while reducing their weight and cost may be gained via the use of FEA, which analyzes the behavior of individual parts and their interactions (Zhou et al., 2018).

## 4 Verifying the FEA with biomechanics *in vitro* tests

Biomechanical testing is a laboratory technique used to analyze bone substitutes’ mechanical characteristics, such as fatigue behavior and endurance life (Johnson and Keller, 2008). The method entails repeatedly loading and unloading the scaffold to mimic the pressures it would face in a living organism. Biomechanical testing is a multi-stage method in which a testing machine is used to provide a regulated load to the scaffold once it has been properly constructed and erected (Ryan et al., 2008). Depending on the test methodology, the load could be administered continuously or cyclically, and its intensity and repetition rate may change (Ban et al., 2020). Information on the developed construct’s deformation, stress and strain distribution, and failure mechanisms is gathered during testing (Zhang et al., 2022). The prepared scaffold could be just an injectable hydrogel, playing a crucial role as a carrier for drug delivery approaches (Goodarzi et al., 2020), or various 2D and 3D scaffolds (Xu et al., 2022). Scaffold mechanical parameters, including stiffness, strength, and fatigue behavior, are calculated after collecting and analyzing this information (Wang et al., 2023).

It is recommended that researchers investigate more about how to make bone substitutes that last longer and function better by conducting biomechanical testing and examining the fatigue behavior of these devices (Wang et al., 2021). For instance, they may determine weak points in the bone structure and devise methods to strengthen them so that they do not give way under cyclic loads. In light of this, biomechanical testing, such as compression, tension, and bending testing, is an invaluable tool for evaluating the mechanical characteristics of bone constructs and refining their design for enhanced performance and longevity (Alaneme et al., 2022).

Given the need for long-term safety in the human body, it is anticipated that metallic cellular scaffolds would have exceptional fatigue characteristics. Zhao et al. (2016) investigated the effect of cell morphology on the compressive fatigue performance of Ti-6Al-4V mesh arrays produced using electron beam melting (EBM). The findings of their study suggest that the fatigue mechanism observed in the three types of meshes (cubic, G7, and rhombic dodecahedron) is primarily attributed to the combined effects of cyclic ratcheting and fatigue crack propagation on the struts. They noted that the presence of a rough surface and the existence of pores inside the struts had a substantial negative impact on the compressive fatigue strength of the struts (Zhao et al., 2016). The authors identified cyclic ratcheting and the start and spread of surface fractures as two fatigue mechanisms of metallic cellular structures (Zhao et al., 2016).

In addition to researching cell transport for bone tissue engineering, Zhao et al. (2010) developed calcium phosphate cement scaffolds that exhibited improved resistance to fatigue and fracture. When reinforced with 15 wt.% chitosan and 20 wt.% polyglactin fibers, the flexural strength of calcium phosphate control in quick fracture rose to 26 MPa, which was much higher than the previous threshold of 10 MPa. The control specimens made of calcium phosphate only reached 5 MPa after being subjected to 2 × 10^6^ cycles of cyclic loading, but the specimens constructed of calcium phosphate-chitosan fiber reached 10 MPa after passing through the loading process. The calcium phosphate-chitosan-fiber failure specimens had an average stress-to-failure of 9 MPa, but the calcium phosphate control had only 5.8 MPa (Zhao et al., 2010). This significant difference in stress-to-failure values was observed. As a result, the authors were able to demonstrate that the constitution of the construct that they had established had an impact on the behavior of weariness.

**FIGURE 2:**
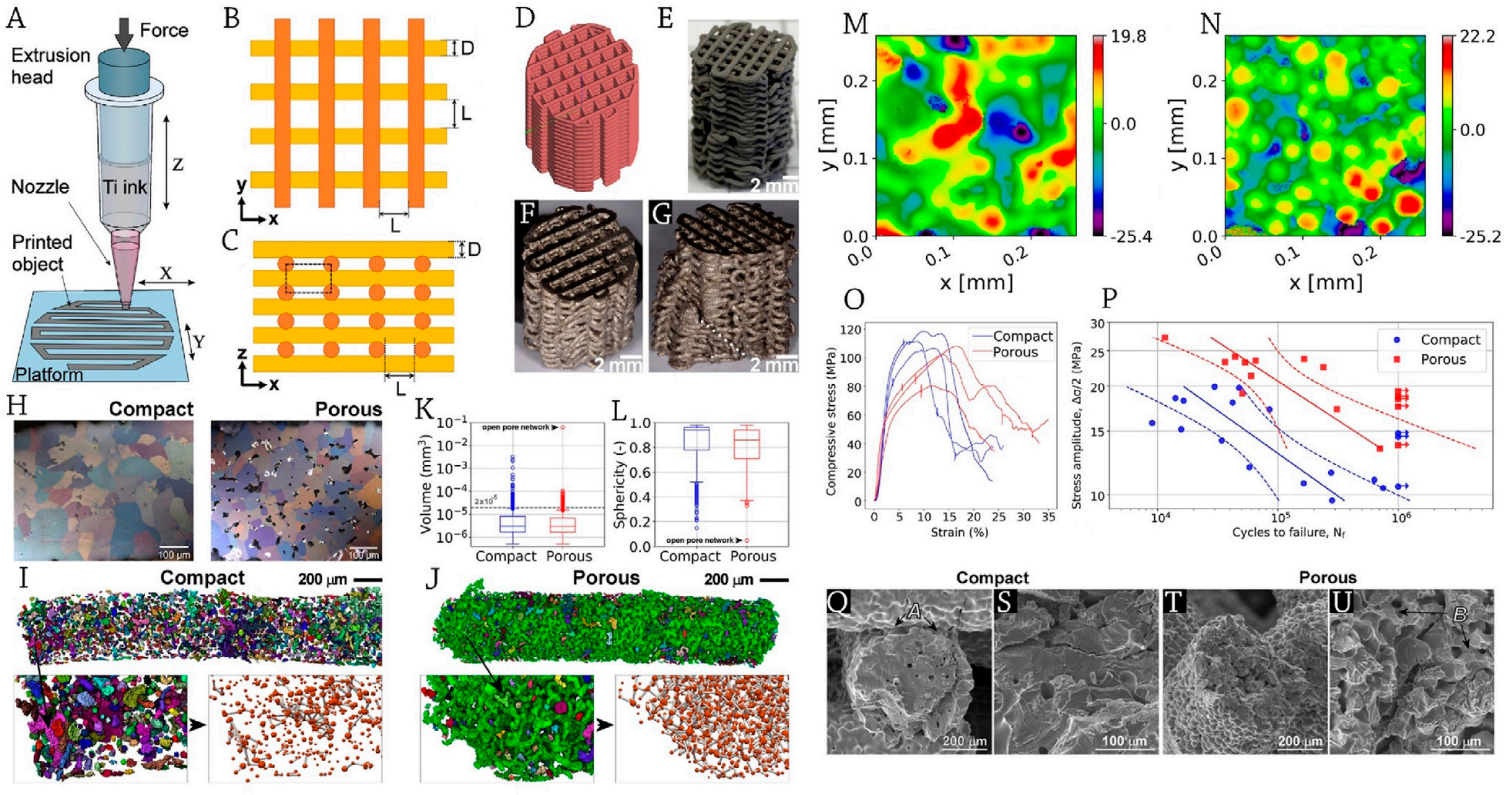
**(A)** Schematic representation of direct ink writing (DIW) process; scaffold design: **(B)** top view, **(C)** lateral view, **(D)** virtual model; The provided photos show representative macroscopic views of **(E)** a titanium scaffold that has been printed and dried, as well as sintered scaffolds **(F)** before and **(G)** after undergoing a fatigue test. The macrocrack is indicated by a dotted line. **(H)** Cross-sections of strands displaying pores and equiaxed alpha Ti grain microstructure; µCT volume depiction of the pore space inside the strands for scaffolds with **(I)** dense and **(J)** porous design. The identified pores are shown using distinct colors. The detailed views in the bottom right show the pores in red and the connections in grey. Box plots illustrate the distributions of **(K)** volumes and **(L)** sphericities of the pores. The images show the surface structures of **(M)** compact and **(N)** porous design scaffolds. The color bar represents the vertical position measured in micrometers. **(O)** The mechanical behavior in compression that is almost static and **(P)** repetitive. The stress-life (S–N) curves are shown with dashed lines representing the 95% confidence interval bands. Displayed are SEM pictures illustrating strand fatigue failure in **(Q, S)** compact, as well as **(T, U)** porous design scaffolds. The arrows are marked to indicate **(A)** a fracture at the junction of the strands and **(B)** secondary cracks at the sintering necks. Reprinted with permission from Slámečka et al., (2023).

Utilizing direct ink writing (DIW) to create titanium scaffolds with hierarchical porosity and human bone-like characteristics, Slámečka et al. (2023) shed light on the high-cycle fatigue behavior of these materials when evaluated under cyclic loads typical of bone implants. Extrusion of a powder suspension (ink) with a high solid content is the basis of their approach (Figure 2A). Sintering is needed to fuse the powder particles and decrease the material’s porosity for the DIW-fabricated components. This means that DIW can construct hierarchically porous structures with pores of varying lengths, such as digitally controlled interstrand pores (often a fraction of millimeters in size) and sintering-state-controlled intra-strand pores (often tens to hundreds of micrometers in size).

The authors reported that cylindrical porous structures measuring 13 mm in diameter and 20 mm in height were constructed using argon-atomized titanium spherical powder that was obtained through sieving (Slámečka et al., 2023). These structures had the same basic base cell design as shown in Figures 2B–E. After meticulously grinding and polishing the top and bottom surfaces of the scaffolds that were eligible for mechanical testing-24 compact and 21 porous-to a height of 14 mm, they conducted quasi-static and fatigue compression tests to verify the necessary flat and parallel surfaces (Figures 2F,G). Also, they used a servohydraulic testing system with a 10 kN load cell and a crosshead speed of 0.5 mm/min to conduct quasi-static testing on three samples per series.

The compactly designed scaffolds exhibited big grains (92 ± 18 μm, Figure 2H) and a limited amount of intra-strand porosity (5.9%), which consisted of tiny, evenly distributed closed pores and isolated pore networks with an uneven shape (Figure 2I), as a result of almost full sintering and noticeable grain development. Grain size was lower (53 ± 11 μm, Figure 2H) and intra-strand porosity was 14.3% in the porous-designed scaffolds that only passed through the middle step of the intermediate sintering state.

They reported that there was a single enormous open-branched pore network (the green pore in Figure 2J) that ran the length of the strand and made up 88% of the intra-strand pore space; a small number of isolated networks with entrance sizes of less than 50 µm made up 7%, and closed pores made up 5% (Figure 2J). In all scaffold types, the pore networks had two to six neighbors per pore, with three being the most common number of connections (Figures 2I,J).

For porous-designed scaffolds, the existence of pore networks caused a long tail to appear in the distributions of pore volume (size) (Figure 2K) and sphericity (Figure 2L), with the furthest outlier indicating an open pore network. The two sintering settings produced distinct scaffold types, one with almost completely dense strands and the other with open network porosity, as confirmed by the noticeable morphological and quantitative variations in intra-strand porosity.

Upon their research, surface morphologies were changed as a consequence of varying sintering conditions. The surface complexity was greater in porous design scaffolds (Sdr = 1.8 ± 0.4) compared to compact design scaffolds (Sdr = 0.6 ± 0.1), according to topographic assessments of the strand surfaces (Slámečka et al., 2023). This was associated with a significantly increased quantity of surface-visible, individually-sintered powder globules (Figures 2M,N).

There was a noticeable difference between the compact and porous design scaffolds in terms of their quasi-static stress-strain responses. The porous design scaffolds exhibited a more gradual rise in stress beyond the elastic regime, as well as a slower drop in load-bearing capacity after achieving maximum strength (Figure 2O). Figure 2P displays the S-N curves for the porous design scaffolds and the compact design scaffolds. Significantly, the findings disprove the commonly held belief that the fatigue life of titanium porous structures improves with decreasing porosity, and the results show that compact design scaffolds withstand fatigue cycling nearly an order of magnitude less than porous design scaffolds. This has important implications for additive manufacturing of porous metallic structures in general.

The two varieties of scaffolds often collapsed because a shear band formed at a 45-degree angle to the loading direction. Figures 2Q–U show that scaffolds with a porous design had much more tortuosity in the fracture route across the strands, but otherwise the fatigue micro-mechanisms were same. The surface/near-surface intra-strand pores and the concave surface remains of the sintering necks were the preferred sites for crack nucleation. At the macroscopic level, the highest theoretical tensile stress zone is located at the intersections of the two perpendicular strands, where many fractures have already begun to form. The strands would sometimes come loose due to fractures that developed around the junctions (Figure 2Q). Figures 2T,U show that porous-designed scaffolds generally had very substantial intra-strand porosity, which allowed cracks to propagate through the sintering necks between particles on the meso-scale (Slámečka et al., 2023).

**FIGURE 3:**
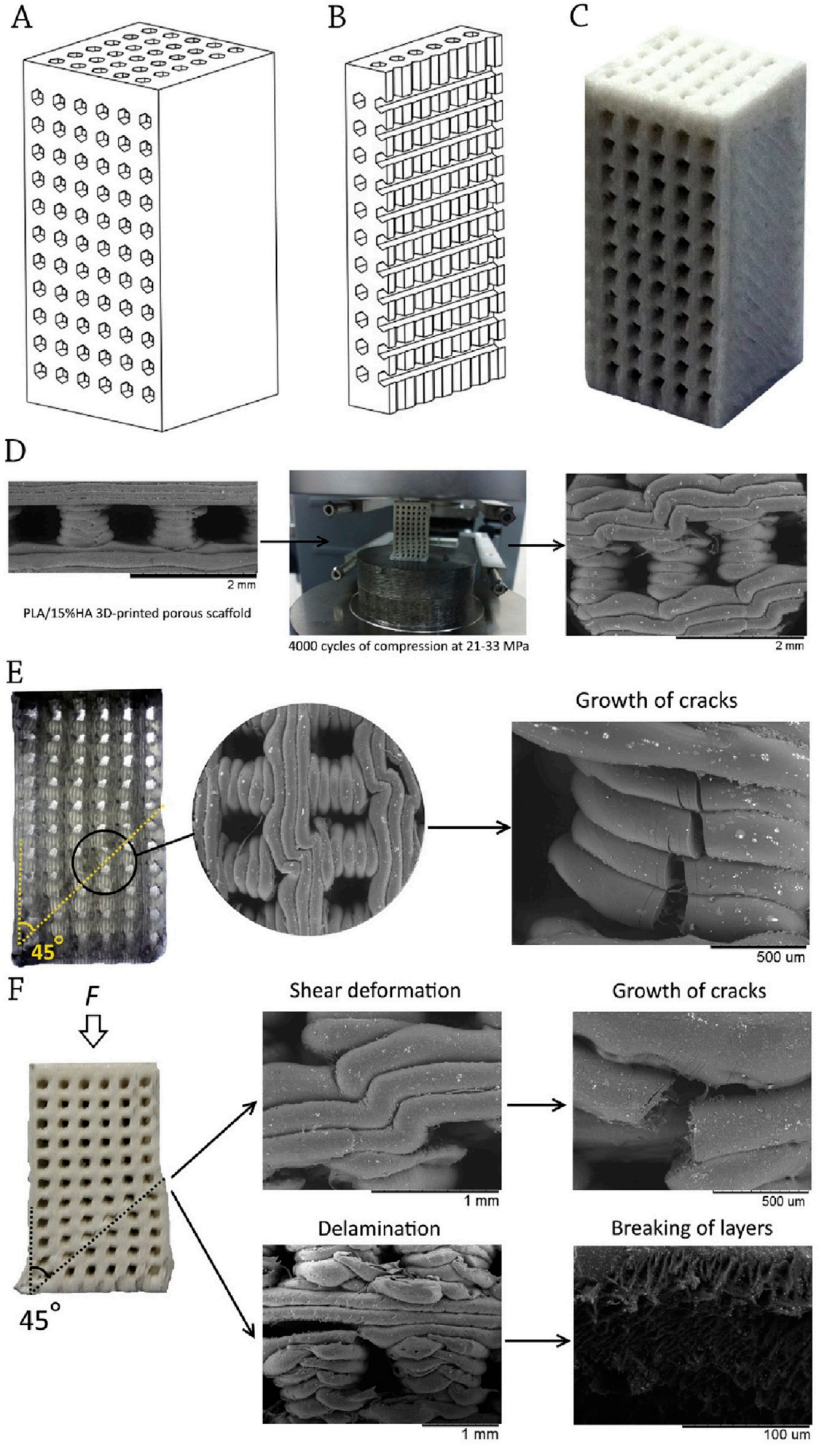
**(A)** Computer-aided designed (CAD) model; **(B)** CAD cut-away model; and **(C)** 3D-printed PLA/HA construct; **(D)** Microstructure of PLA-based porous bone construct before and after cyclic loading; **(E)** Structure of PLA porous bone construct after 4,000 cycles loading; **(F)** Structure of PLA/HA porous bone construct after 4,000 cycles loading; Reprinted with permission from Senatov et al. (2016).

Senatov et al. (2016) investigated the effects of polylactic acid (PLA) and PLA-blended with 15 wt.% hydroxyapatite (HA) employing a technique called fused filament manufacturing. Figures 3A–C depict the computer model, the cut-away model, and the bone constructs. They claimed that the standard pore size for scaffolds is 700 μm, and their porosity is 30% by volume. Researchers examined the 3D porous scaffolds’ modulus of elasticity, sample height, elastic deformation, stored energy, and structural properties during low-cycle testing (Senatov et al., 2016).

When the load was increased, the micropores inside the printed layers collapsed, and the scaffold portions distorted locally. As seen in Figure 3D, fatigue caused little fractures to appear in the flexing layers, which subsequently grew into a larger one and eventually brought down the whole structure. Fracture resistance was higher in the early stages of cyclic loading for PLA/HA porous scaffolds. Cyclic loadings inhibited the formation of cracks in HA particles (Senatov et al., 2016).

Averett et al. (2011) performed fatigue tests at 3 and 30 Hz and discovered that when ceramic nanoparticles were added to PLA/nanoclay, the materials behaved similarly. The material was destroyed because of the way in which the shift occurred. The crack was spreading at an angle of 45° to the direction of the applied force. Figures 3E,F show that the destruction often started around the pillar (bottom left corner of the sample).

Researchers found that cyclic loading led to crack development, crack propagation, loss of height, pore collapse, delamination, bending, and shear in the printed layers. The inclusion of dispersed HA particles decreased the rate of defect accumulation. A stronger fracture resistance was found for PLA/15%HA porous scaffolds during cyclic loading at a stress of 21 MPa, suggesting their potential use as implants for trabecular bone replacement (Averett et al., 2011).

In order to achieve the requirements of long-term bone implantation, Liu et al. (2023) offers a selective approach to finding the optimal structural form and fatigue life for implanted scaffolds. The scaffolds were made using additive manufacturing EBM using Ti-6Al-4V after a new mathematical technique was used for their design. Scaffolds with a fatigue life more than 10^6^ at a stress level of 0.2 were guaranteed after Micro-CT, FEA, and SEM showed the failure mechanism of the scaffolds under high cycle fatigue. Results showed that fatigue life was improved without a compromise in porosity when scaffolds with bigger unity size helped to limit notch locations for crack initiation.

**FIGURE 4:**
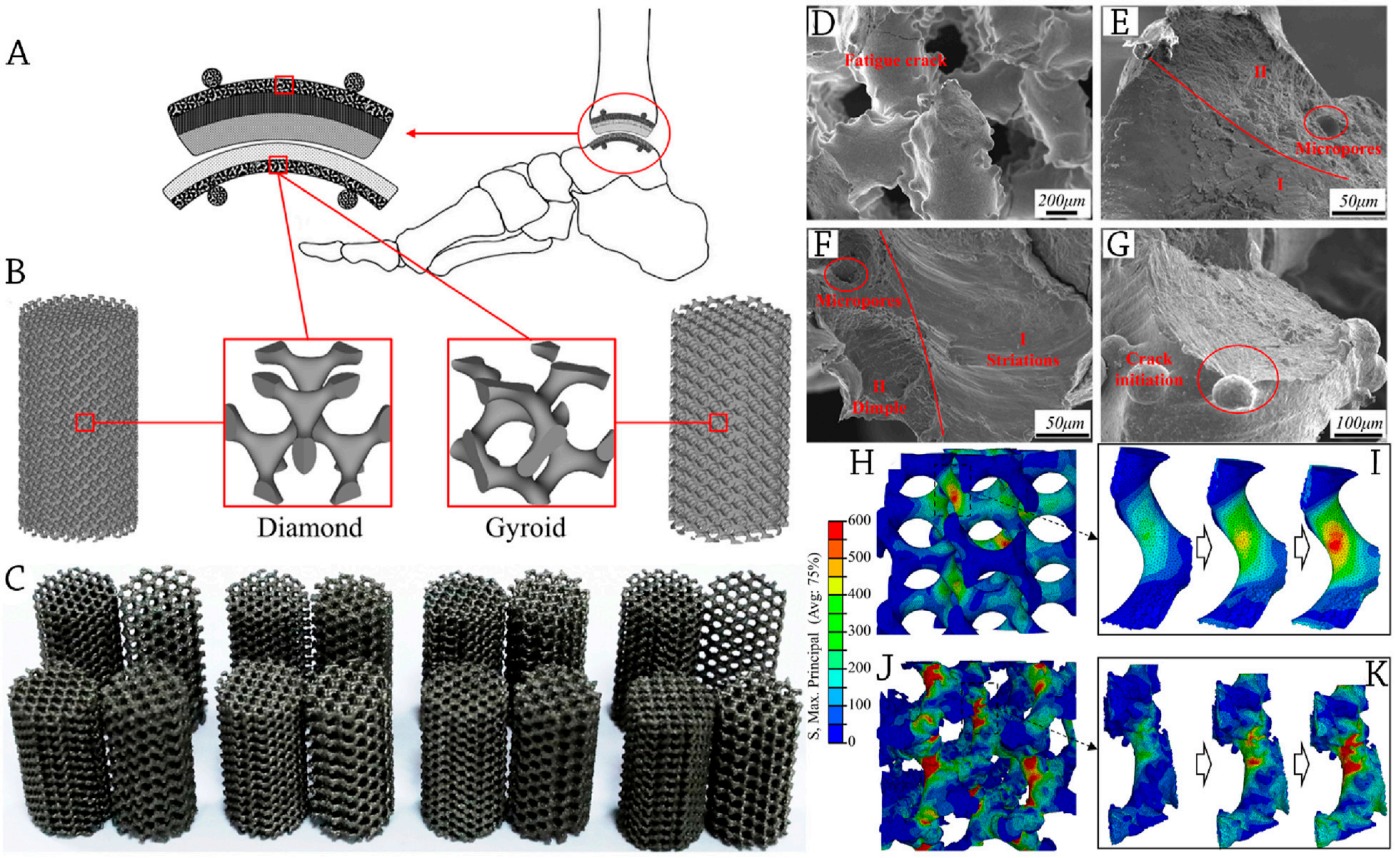
**(A)** A description of a prosthetic device for the foot and ankle that includes bionic porous scaffolds, which serve both biological and biomechanical purposes; **(B)** The prosthetic device incorporates specially designed porous scaffolds; **(C)** The scaffolds are created using Electron Beam Melting (EBM) and are tested for compression and fatigue resistance. The SEM was used to examine the fracture morphologies of the scaffold after fatigue testing; **(D)** Struts exhibiting fatigue fractures; **(E, F)** A close examination of the fracture section reveals two distinct morphologies. The first morphology, labelled as I, consists of striation areas that indicate the beginning and propagation of the crack. The second morphology, labelled as II, is characterized by dimple regions where the crack ultimately breaks. **(G)** Initiation of cracks from surface irregularities. **(H–K)** The stress distribution of the modelled scaffold is visualized using colored patterns in a FEA, highlighting the progression of maximum primary stress. The designed model exhibits a more homogeneous distribution of stress. The CT-reconstructed model reveals that stress is localized at the base of the uneven surface. Reprinted with permission from Liu et al., (2023).

At the interface between the prosthesis and human bone, bionic porous scaffolds enable the migration of nutrients and bone ingrowth, and they deliver compatible biomechanics to support the body’s weight with compression loads. This allows for ankle joint movement, as shown in Figure 4A. The continuous surface was built using two kinds of scaffold units, gyroid (G) and diamond (D), which were created using the MATLAB R2020a program using generic STL files. Models of porous scaffolds D and G, with their corresponding unit cells indicated, are shown in Figure 4B. The samples created by EBM for studies, such as Micro-CT and mechanical testing, are shown in Figure 4C.

Studies showed that the rough surfaces with bonded powder are the origin of fatigue fractures in scaffolds (Xiong et al., 2020). Figures 4D–G show a similar phenomenon, with radial flow striation radiating from the bonded powder’s root to the struts’ center. After that, a striped streamline form was subtly created via the process of long-term cycling.

A crucial component of fatigue damage to scaffolds is the local buckling of rough surfaces (Hou et al., 2022). Results showed that modeled scaffolds demonstrate a consistent distribution of stress at a load condition of 0.4 stress level. The CT-reconstructed scaffolds, on the other hand, shows that the rough surface causes a significant concentration of stress in abrupt bends. At the critical site of crack nucleation and initiation, as seen in SEM fracture morphologies of Figures 4D–G, the FEA results likewise exhibit a prominent notch effect with significant roughness. The coupling forces of buckling and bending dictate the deformation of the struts in most AM-fabricated cellular structures (Dave and Gomes, 2019), as shown in Figures 4H–K.

## 5 Fatigue behavior models for bone substitutes

A variety of models and mathematical formulae may be used in order to define the fatigue behavior and durability of bone replacements. These models and formulas are dependent on the material attributes, design, and loading conditions of the bone substitute products.

### 5.1 Coffin-Manson model

Metal and polymer fatigue lives may be predicted using the Coffin-Manson model approach (Putra and Machmud, 2020). The Coffin-Manson equation establishes a relationship between the strain range (Δε) of a material and the number of load cycles (N) needed to cause failure at a given stress amplitude (σ). This equation Equation 3 can be adjusted to take into account the elastic modulus, strain, and pore size distribution of bone constructs.

$$N={\frac{1}{\Delta \varepsilon }}^{b}\times {\frac{\sigma }{\sigma_{f}}}^{-c}$$ (3)

Where the fatigue strength of the material is $\sigma_{f}$ , and b and c are constants. The model presupposes that there is a linear relationship between the logarithm of the strain range and the logarithm of the number of cycles before failure (Bakkar et al., 2021). The lifetime of a material under cyclic loads may be predicted with the help of this model. It is possible to predict the number of failure cycles given information about the strain range and fatigue strength of the material (Bakkar et al., 2021). With this data in hand, engineers can create long-lasting products that are up to code. It is possible that the model may be used to represent bone substitutes in medical contexts, despite its more common usage in the design of metal and polymer components for aerospace, automotive, and biomedical applications (Abraham and Venkatesan, 2023).

**FIGURE 5:**
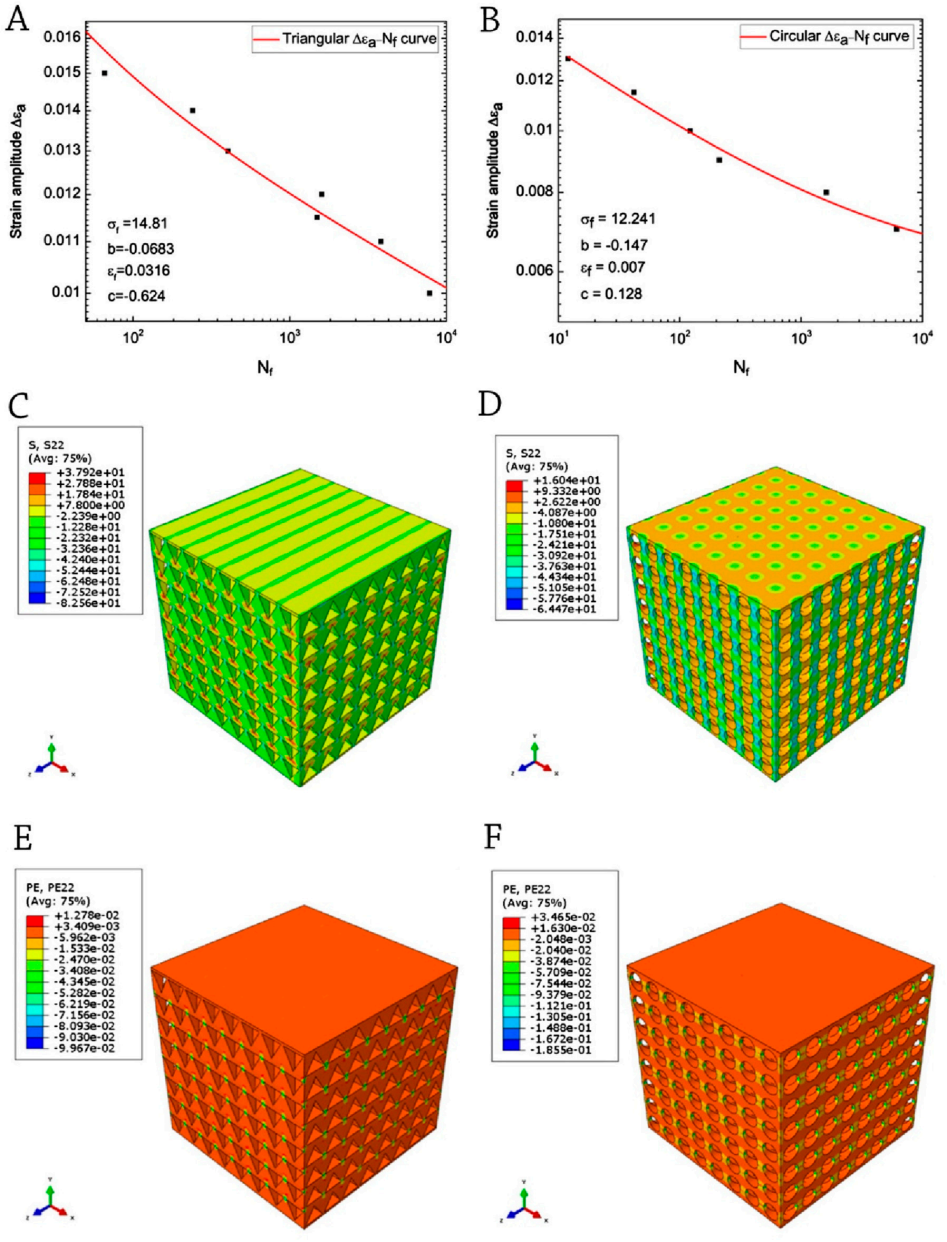
Coffin-Manson relation-fitted ΔƐ_a_–N_f_ curves: **(A)** a sample of triangle-shaped pores; **(B)** a sample of circles-shaped pores. Uniaxial compressive test simulations: **(C)** stress distribution of triangular-pored PLA scaffolds; **(D)** stress distribution of circular-pored PLA scaffolds; **(E)** plastic strain distribution of triangular-pored PLA scaffolds; and **(F)** plastic strain distribution of circular-pored PLA scaffolds; Reprinted with permission from Gong et al. (2017).

Gong et al. (2017) conducted a study to investigate the low-cycle fatigue characteristics of 3D-printed PLA scaffolds with a porosity of 60% and two distinct pore geometries. The study employed strain-controlled loading and utilized the Coffin-Manson model for analysis (Figures 5A,B). Both types of specimens’ experience strain softening following the initial cyclic hardening. The bone constructs possessing circular pores demonstrate a consistent ability to withstand fatigue-induced damage, making them an advantageous choice for bone repair applications. With respect to the accrual of inelastic deformation, it can be observed that the triangular scaffold exhibits a higher degree of sensitivity to cyclic loading. The enhanced fatigue properties of the scaffold containing circular pores can be attributed to the uniform distribution of mechanical stress and the reduction of stress concentration resulting from the incorporation of circular pores (Figures 5C–F).

### 5.2 The Weibull distribution

The Weibull distribution is a useful statistical model for describing the probability distribution of a material’s or component’s fatigue life (Pedrosa et al., 2020). There is a correlation between the number of load cycles (N) and the chance of failure for a given stress amplitude (σ) in the Weibull distribution function (Pedrosa et al., 2020). Bone constructs’ susceptibility to failure under different stress conditions may be predicted using this approach Equation 4.

$$P_{\text{failure}}=1-e^{-{\frac{N}{N_{0}}}^{m}}$$ (4)

N represents the number of load cycles required for failure, whereas $N_{0}$ is an experimental constant that indicates the number of cycles needed for failure in 63.2% of the specimens. The Weibull modulus, denoted as m, characterizes the form of the probability distribution function (P_failure_) (Pedrosa et al., 2020).

The Weibull distribution was found to be helpful in evaluating the durability of bone scaffolds subjected to cyclic loading (Luis et al., 2019; Heidari et al., 2020). If researchers have access to the fatigue life distribution function of the scaffold, they may forecast the probability of failure after a certain number of loading cycles. They perform fatigue tests on a group of bone substitutes, documenting the number of cycles until failure for each sample. They then use statistical techniques to get the values for $N_{0}$ and m required to fit the data into the Weibull distribution function (Mahmoud et al., 2021). Bone substitutes can be made better with the help of the Weibull distribution, which can direct the selection of materials and the geometry of the scaffold. If the probability of failure increases with the number of loading cycles, as indicated by the distribution function, then the design can be modified to reduce the likelihood of fatigue failure (Wilkie and Galasso, 2021).

Prakash et al. (2021) aimed to examine the mechanical reliability and *in vitro* bioactivity of porous scaffolds made from a combination of HA and PLA using 3D printing technology. Their experiments were conducted to investigate the impact of varying weight percentages of HA in a PLA matrix, infill density, and post-printing thermal stimulus on the flexural and compressive strength. Furthermore, the optimal selection of input parameters was identified in order to fabricate the test specimens for reliability analysis based on the observed mechanical properties.

They reported that this was accomplished by employing the Weibull distribution. Additionally, an investigation was conducted on the fracture morphology of the porous scaffolds made from PLA/HA. Their study’s findings underscore the significant influence of processing parameters on the mechanical properties of 3D-printed porous scaffolds. Moreover, the *in vitro* examination demonstrated exceptional growth, proliferation, and differentiation of osteoplastic cells. The authors claimed that, in addition to these findings, the Weibull distribution analysis supports the notion that the printed porous scaffolds exhibit mechanical reliability (Prakash et al., 2021).

### 5.3 The miner’s rule

Miner’s rule is an empirical model commonly used to estimate the wear and tear on a material or component after repeated loading and unloading (Hectors and De Waele, 2021). The rule is based on the premise that repeated loadings cause cumulative damage until a material or component fails. Bone scaffolds can have their fatigue life estimated using Miner’s rule under a variety of loading scenarios. The rule gives a ballpark figure Equation 5 for how many times a material may be loaded and unloaded before breaking. The cumulative damage that is caused by the load cycles is represented by the symbol ΣN_i_, where N_i_ represents the number of loading cycles that have occurred at stress level i, f represents the fatigue life of the material at stress level i, and N_i_ represents the number of stress levels (Li et al., 2023). The N_i_/N_i, f_ parameter is calculated by dividing the total number of loading cycles by the lifespan of the material at that particular stress level. According to the rule, a material or component will fail due to cumulative damage if the sum of the ratios between the number of loading cycles and the number of cycles to failure for each stress level is more than 1. This rule applies to all stress levels.

$$\sum \frac{N_{i}}{N_{i,f}}\ll 1$$ (5)

The fatigue life of a bone structure may be estimated using Miner’s rule for a range of loading circumstances. The scaffold should not be used for the application if the expected number of loading cycles is less than or equal to the number of cycles that are expected to harm the bone replacement. On the other hand, Miner’s rule makes the erroneous assumption that every loading cycle is equally harmful. Using the Coffin-Manson model or the Weibull distribution in conjunction with Miner’s rule may provide a more accurate estimate of the fatigue life of bone scaffolds (Jiang et al., 2022).

## 6 Future perspectives

With a better understanding of the fatigue characteristics of load-bearing bone scaffolds, it is possible to optimize the design, material selection, and fabrication of bone structures to meet the specific needs of individual patients. This notion revolves around the production of implants that accurately correspond to an individual’s anatomical and biomechanical needs. Paying close attention to fatigue qualities paves the path for custom-made implants that excel in both structural optimization and fatigue resistance.

Implementing this technique can effectively decrease the occurrence of implant failures, mitigate problems, and enhance patient comfort and overall quality of life. Moreover, the incorporation of cutting-edge manufacturing methods, such as 3D printing, offers the potential to create patient-specific scaffolds with precisely tailored fatigue-resistant characteristics. The integration of technology and the study of fatigue enables the achievement of optimal performance and lifespan tailored to the specific requirements of each patient.

As mechanical performance of the scaffold and cell surrounding niche plays a crucial effect in tissue regeneration and repairing procedure (Mirabdali et al., 2024), it is anticipated that developing new FEA models for predicting loading and unloading cycles in bone scaffolds could results in predicting of osteogenesis and the success of bone structures. In addition, investigating the fatigue characteristics of bone scaffolds also facilitates the exploration of broader applications in clinical settings and the attainment of regulatory endorsement. As our comprehension of scaffold fatigue resistance improves, we may establish standardized testing methodologies to guarantee the safety and efficacy of these materials in load-bearing applications. This accelerates the process of obtaining regulatory approval, hence increasing the availability of new scaffold designs for widespread clinical utilization. Moreover, possessing comprehensive fatigue data can enhance the assurance of healthcare practitioners and regulators, hence fostering broader recognition of bone scaffolds as feasible substitutes for conventional materials.

## 7 Conclusion

The fatigue performance of bone scaffolds is a crucial determinant in their successful clinical use for bone regeneration and substitution. The mechanical properties of bone scaffolds are greatly influenced by a range of parameters, including structural characteristics such as pore size, interconnectivity, and distribution, as well as material variables including composition, degradation rate, and manufacturing process. To create strong and dependable procedures for engineering bone tissue, it is necessary to have a thorough grasp of the elements that determine how these scaffolds withstand fatigue. Improving the fatigue strength of bone scaffolds may be achieved by selectively choosing materials and adjusting mechanical parameters. This can result in favorable healing results.

The endurance limit and fatigue strength of bone scaffolds are controlled not only by the mechanical characteristics of the implant, but also by the quality of the surrounding tissue and the physiological factors of the host. The fatigue behavior of a scaffold is also heavily influenced by its implantation location, particularly the bone quality, microarchitecture, and stress loading in that area. The microstructure, porosity distribution, interconnectedness, and pore size of the scaffold have all been shown to affect its fatigue life. Scaffolds with large pore sizes and limited interconnectivity may experience delayed failure due to fracture propagation. On the other hand, scaffolds with minimal pore size and high interconnectivity exhibit extended fatigue life by resisting fracture propagation. Therefore, considering various aspects such as structural and material qualities, surrounding tissue quality, host physiological parameters, and implantation location is crucial in understanding and improving the fatigue behavior of bone scaffolds, ultimately leading to the development of biocompatible scaffolds with superior endurance limits and fatigue performance.
